# A Review of Nanowire Devices Applied in Simulating Neuromorphic Computing

**DOI:** 10.3390/nano15100724

**Published:** 2025-05-11

**Authors:** Tianci Huang, Yuxuan Wang, Zhihan Jin, Hao Liu, Kaili Wang, Tan Leong Chee, Yi Shi, Shancheng Yan

**Affiliations:** 1 School of Integrated Circuit Science and Engineering, Nanjing University of Posts and Telecommunications, Nanjing 210023, China; 1223228117@njupt.edu.cn (T.H.); 1223228118@njupt.edu.cn (Z.J.); 2023221102@njupt.edu.cn (H.L.); 1222228419@niupt.edu.cn (K.W.); cheelong@gmail.com (T.L.C.); 2 National Key Laboratory of Solid-State Microwave Devices and Circuits, Nanjing 210005, China; 3 School of Electronic Science and Engineering, Nanjing University, Nanjing 210023, China

**Keywords:** nanowire devices, neuromorphic computing, neural network

## Abstract

With the rapid advancement of artificial intelligence and machine learning technologies, the demand for enhanced device computing capabilities has significantly increased. Neuromorphic computing, an emerging computational paradigm inspired by the human brain, has garnered growing attention as a promising research frontier. Inspired by the human brain’s functionality, this technology mimics the behavior of neurons and synapses to enable efficient, low-power computing. Unlike conventional digital systems, this approach offers a potentially superior alternative. This article delves into the application of nanowire materials (and devices) in neuromorphic computing simulations: First, it introduces the synthesis and preparation methods of nanowire materials. Then, it analyzes in detail the key role of nanowire devices in constructing artificial neural networks, especially their advantages in simulating the functions of neurons and synapses. Compared with traditional silicon-based material devices, it focuses on how nanowire devices can achieve higher connection density and lower energy consumption, thereby enabling new types of neuromorphic computing. Finally, it looks forward to the application potential of nanowire devices in the field of future neuromorphic computing, expecting them to become a key force in promoting the development of intelligent computing, with extensive application prospects in the fields of informatics and medicine.

## 1. Introduction

With the rapid development of artificial intelligence and brain-like computing fields in recent years, researchers have put higher demands on building more intelligent and efficient computing systems [1]. Traditional computing systems face high energy consumption and low computational efficiency when dealing with complex neural network tasks. This has prompted the search for new computing models and devices to simulate the structure and function of the human brain’s neural networks [2,3,4]. For example, a novel synaptic device architecture, the ion-gated vertical transistor (IGVT), has recently been successfully implemented and promptly applied to implement brain-like perception such as artificial vision, touch, taste, and hearing. Within such a short time frame, IGVT has already demonstrated faster data processing speeds and more promising storage capabilities compared to many traditional neuromorphic devices, even when operating at lower voltages and consuming less power [5].

Implementing physical devices with synaptic characteristics is key to building the hardware foundation for neuromorphic computing systems and is decisive for advancing the field. Initially, the operations of synapses were emulated using complementary metal–oxide–semiconductor (CMOS) technology in neuromorphic engineering. However, these CMOS circuits consume much more energy than biological synapses, making it difficult to scale the circuit size to a level comparable to that of the brain. In subsequent research, inspired by biology, researchers have explored resistive switching memory, memristors, and atomic switches in neuromorphic circuits and have studied important synaptic learning rules, such as spike-timing-dependent plasticity (STDP) and the transition from short-term memory to long-term memory.

As a cutting-edge technology, nanotechnology offers new possibilities for brain-like computing [6,7,8,9,10]. Nanowire devices, as an important component of nanotechnology, play a significant role in advancing the field [11,12]. Possessing excellent electrical, optical, and magnetic properties, they can simulate the characteristics of neurons and synapses, providing new ideas and methods for the construction of brain-like computing systems [13,14,15,16,17].

Due to their small size, nanowire devices can achieve high-density integration within a limited space, which is crucial for simulating the vast number of neurons and synaptic connections in the brain. In these nanoscale ion/electron hybrid two-terminal artificial synapses, ion migration/diffusion, the formation of conductive channels, and electrochemical reactions are commonly used to simulate neural activity. This high-density integration helps to improve computational efficiency and reduce power consumption. Moreover, nanowire devices are flexible and can adapt to various application scenarios, including wearable devices and flexible electronics. This flexibility gives nanowire devices an advantage in simulating biological neural networks, enabling better simulation of the brain’s dynamic changes and plasticity. Finally, nanowire devices typically have low power consumption when simulating synaptic functions. For example, as a nanowire device, memristors change their resistance with the current passing through them and can “remember” previous current values. This characteristic gives memristors the advantage of low power consumption when simulating synaptic functions. In summary, nanowire devices have significant advantages in simulating brain-like computing, including high-density integration, flexibility, low power consumption, and synaptic function modeling. These advantages give nanowire devices broad development prospects in brain-like computing.

**Figure 1:**
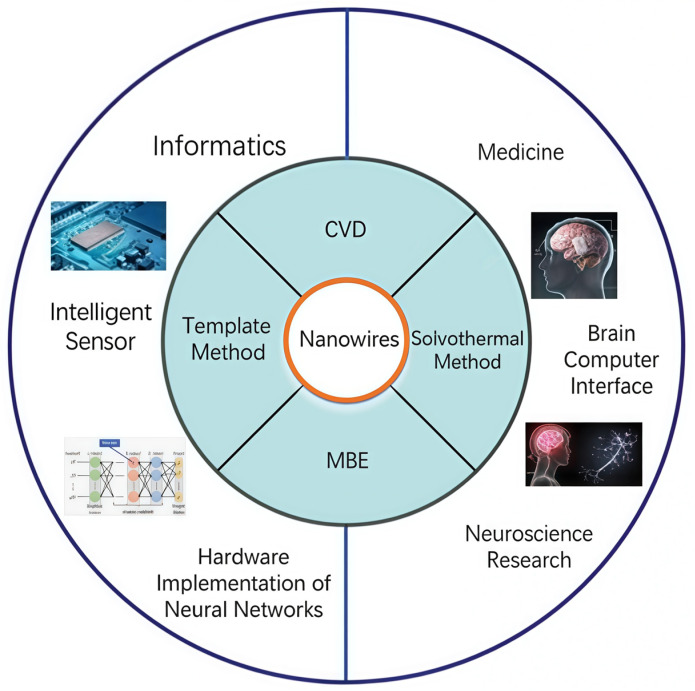
Synthesis methods and application fields of nanowires.

This article primarily introduces the commonly used synthetic methods for the preparation [18] of nanowires and nanowire devices simulating synaptic characteristics [19,20]. Figure 1 illustrates common nanowire synthesis techniques, such as chemical vapor deposition (CVD) and solvothermal method. These methods can produce nanowires with different sizes, shapes, and compositions to meet various application requirements. Research on simulating synaptic characteristics with nanowire devices mainly focuses on their nonlinear conductance characteristics, charge storage capacity, and rapid response times, all essential for achieving efficient neuromorphic computing.

Looking forward to the future [21,22], the potential applications of nanowire devices in medicine and informatics are extensive. For example, nanowire devices can be used in brain–computer interfaces to build high-density, high-sensitivity electrode arrays to achieve efficient communication with the nervous system. Regarding smart sensors [23], nanowire devices’ high sensitivity and rapid response characteristics make them ideal for detecting biomarkers and environmental changes.

The application prospects of nanowire devices in brain-like computing are vast [24,25]. They can improve computational efficiency, reduce energy consumption, and drive the innovative development of a new generation of intelligent systems [26]. With the deepening of research and the advancement of technology, it can be anticipated that nanowire devices will play an increasingly important role in future technological revolutions.

In summary, through a review of the synthesis methods of nanowire devices and their applications in simulating synaptic characteristics, highlights the significant advantages of nanowire devices in brain-like computing and envisions their broad future application prospects. These studies not only provide new insights for addressing the limitations of traditional computing systems but also lay a solid foundation for promoting the innovation and development of brain-like computing systems.

## 2. Nanowire Fabrication

Nanowire devices are electronic devices based on materials prepared at the nanoscale, characterized by high structuring and controllability, typically composed of conductors or semiconductor materials at the nanoscale [18]. These devices can be used for computing, storage, and other applications, and they show great potential in brain-like computing.

### 2.1. Chemical Vapor Deposition

CVD for growing nanowires utilizes gas-phase chemical reactions under high temperatures, plasma, or laser assistance. The formation of nanomaterials through nucleation and growth can be directed by adjusting factors such as pressure, gas flow rate, and substrate temperature. This method of production is characterized by its ease of use, affordability, high crystallinity, and purity [18,27]. The shape of metal nanowires can be manipulated by altering parameters like the metal precursor, substrate material, and annealing temperature.

Temperature is one of the most critical factors affecting the growth of nanowires in the CVD process. Different material systems and desired nanowire properties may require different temperature ranges. For example, in the preparation of iron nanowires, the decomposition temperature of ferrocene is around 600 °C, which is crucial for releasing iron atoms from the gaseous precursor and depositing them onto the substrate to form nanowires. Parameters such as pressure and gas flow rate affect the distribution and concentration of gaseous precursors in the reaction chamber, influencing the nanowires’ uniformity and density. Appropriate pressure and flow rate help achieve a uniform distribution of precursors, avoiding aggregation or uneven diameter of nanowires due to localized high concentrations. The substrate not only provides support but may also participate in catalytic reactions or affect nanowires’ growth direction and arrangement.

In some cases, such as the preparation of nickel nanowires, special substrate surface treatment (like forming a silicon-based film) is crucial for nanowire growth. The use of catalysts affects CVD; although nanowires can be grown without catalysts in CVD, in some applications, using catalysts can better control the diameter, length, and growth location. The type and amount of catalysts need to be optimized based on the target material and desired performance.

**Figure 2:**
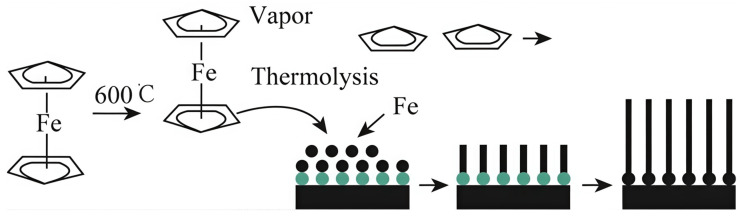
Preparation of single crystal iron nanowires by CVD [28]. Copyright 2015, Elsevier B.V.

CVD is widely used in the fabrication of nanowires. For example, Hu et al. [28] used ferrocene as a metal source and alumina as a substrate to prepare single-crystal iron nanowires with diameters greater than 125 nm and lengths of about 2 μm through CVD. Figure 2 illustrates the synthesis mechanism: Upon heating, ferrocene evaporates and moves to the core heating area; at 600 °C, it breaks down into iron and cyclopentadiene. The iron atoms then settle on the substrate, alloying with gold nanoparticles that act as catalysts, creating sites for iron nanowire nucleation. Ultimately, iron nanowires initiate and expand from these nucleation points. It should be noted that, in the CVD preparation process, metal nanowires can grow without external catalysts. Chan et al. [29] used CVD to prepare single-crystal nickel nanowire arrays (diameters ranging from 50 to 300 nm, approximately 5 μm in length) on substrates composed of amorphous silica and silicon without the addition of external catalysts. The experiments showed that adjusting the heating temperature could control the diameter and growth density of the nickel nanowires. To rationally explain the growth phenomenon, the team proposed a growth mechanism for the nickel nanowires [30]: First, chlorine gas produced from the heating process (below the deposition temperature) of nickel hexahydrate reacts with the substrate, causing the substrate to crack and release silicon atoms; then, a small number of silicon atoms form silicon clusters at fixed positions on the silica surface, which further form a silicon-based film; finally, when the temperature rises to 650 °C, nickel atoms begin to deposit, and due to the presence of the silicon-based film, the nickel nanowires grow at the silicon–nickel interface.

### 2.2. Template Method

The template method typically utilizes nanostructured pores to control the growth of nanomaterials, thereby facilitating the preparation of nanowires. This approach can precisely regulate nanowires’ morphology, structure, and size and improve their dispersibility. Therefore, the template method is crucial for preparing nanowires. Common templates in liquid-phase synthesis systems include porous materials, self-assembled molecular structures, and biological macromolecules.

Porous material templates, such as anodized aluminum oxide (AAO) membranes or polycarbonate membranes, are often combined with liquid-phase deposition methods to prepare metal nanowires. AAO templates have the advantages of uniform pore distribution, high pore density, and artificially controllable pore size and length. Preparing metal nanowires involves template preparation, electrochemical deposition, and template dissolution. The dimensions and shape of metal nanowires can be regulated by modifying operational parameters including the voltage applied, current density, and the concentration of the metal precursor.

**Figure 3:**
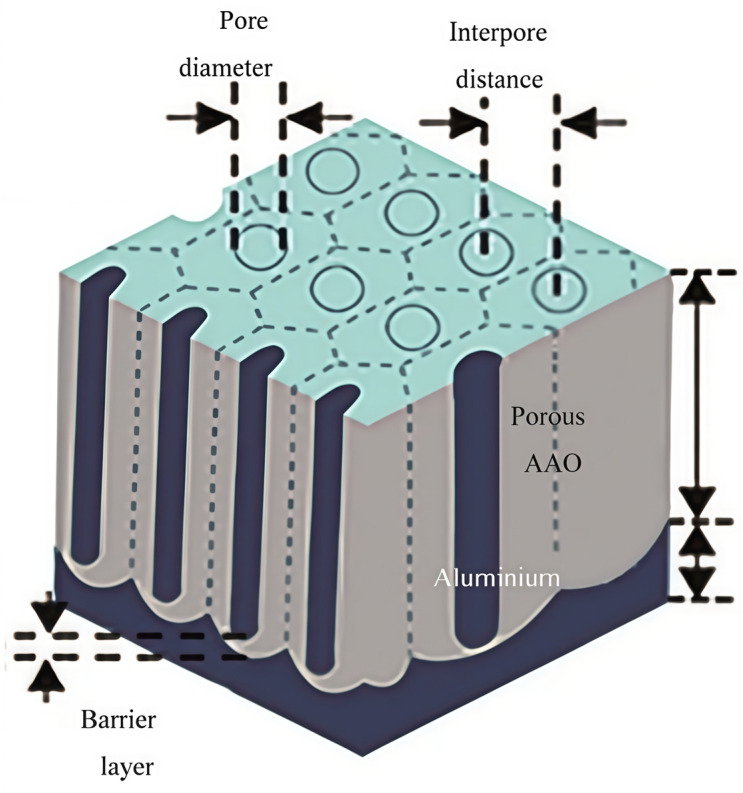
Schematic diagram of the AAO template [31]. Copyright 2019, American Chemical Society.

In preparing AAO templates, high-purity aluminum foils that have been polished are typically used as the anode and placed in an electrolytic cell containing an acidic electrolyte. After applying a specific direct current voltage, the electrolysis process is carried out, and by adjusting parameters such as electrolysis time, AAO templates are formed, as shown in Figure 3. However, these templates are not directly applicable to preparing metal nanowires and usually require treatments such as pore widening and conductive layer coating. During the electrochemical deposition process, the AAO template serves as the cathode in the electrolyte. Under the influence of an electric field, metal ions migrate to the vicinity of the cathode and undergo electrochemical reactions, forming nuclei and depositing at the bottom, thus creating metal nanowires. In the template dissolution stage, alkaline solutions such as sodium hydroxide typically dissolve the AAO template, yielding metal nanowires.

AAO templates are relatively commonly used in the template method for preparing one-dimensional nanomaterials [31]. In recent years, the AAO template method has been continuously developing and improving, with one advancement being the simplification of pre-treatment operations, such as removing the barrier layer before use. Ganapathi et al. [32] thinned the barrier layer of the AAO template. They exposed the underlying aluminum metal through chemical etching, preparing uniformly sized copper nanowires (with a diameter of about 73 nm and a length of about 6 μm) without metal film deposition. To enhance the flexibility of the AAO template method, Guiliani et al. [32] invented a method for transferring AAO templates with a double polymer protective layer, allowing the direct fabrication of segmented metal nanowires on both planar and curved substrates using the AAO template method.

Additionally, Wen et al. [33] designed the pores of the AAO template so that both circular-sectioned nickel nanowires and square-sectioned silver nanowires could be produced simultaneously within the same template. Although AAO templates can make the shape of metal nanowires more uniform, the template size limits the aspect ratio and production scale of metal nanowires. Furthermore, the brittleness of AAO templates makes them prone to damage during use, and the complexity of the preparation steps, to some extent, restricts the widespread application of AAO template technology.

Overall, the variety of porous material templates is still relatively limited, and most are disposable, making them difficult to reuse. This is mainly because the method requires high uniformity of the template pore size and ease of removal. In addition, nanowires synthesized using this method typically exhibit a polycrystalline structure, and the template size limits their yield. In the future, further simplifying the preparation process of the template method and developing templates with large-scale production capabilities and reusability will be the direction of development.

**Figure 4:**
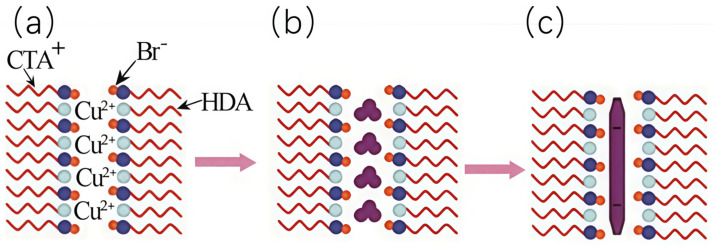
Schematic diagram of the preparation of copper nanowires by liquid crystal template method [34]. Copyright 2012, American Chemical Society.

Self-assembled molecular templates often pertain to the structured assemblies created by surfactants, including micellar and liquid crystalline phases. This template method can control nanomaterials’ nucleation and growth processes by adjusting parameters such as surfactant concentration, reaction temperature, reaction time, and the reducing agent, thereby regulating the morphology and structure of nanowires. The preparation process mainly includes three steps, as shown in Figure 4: a. Surfactant self-assembly: above the critical micelle concentration, surfactants form stable rod-like micelles. b. Nanowire growth: metal sources undergo reduction reactions within the micelles, forming nanowires. c. Removal of surfactants: pure metal nanowires are obtained by eliminating surfactants through solvent extraction or thermal treatment. The advantage of this method is the precise control over the morphology and structure of nanowires. In the future, further optimizing the preparation process and improving yield and purity will be key directions for developing this method.

Compared to porous material templates, the preparation process of self-assembled molecular structure templates is relatively simple, and they can be easily removed by solvent washing, making them widely used in the preparation of metal nanowires.

### 2.3. Solvothermal Method

The solvothermal method is a process that enables the crystallization and growth of materials from solutions of soluble metals or metal–organic salts under high-temperature and -pressure conditions. In a typical synthesis process, a metal source, solvent, reducing agent, and reagents that guide crystal growth [such as polyvinylpyrrolidone (PVP), oleylamine (OLA), etc.] are added to an autoclave in certain proportions. Subsequently, the reaction is carried out under subcritical or supercritical conditions, forming metal nanowires. This method is highly controllable and scalable, allowing for preparing nanomaterials with excellent properties. In the future, further improving the preparation efficiency and optimizing the morphology and structure of nanowires will be key directions for developing this method.

**Figure 5:**
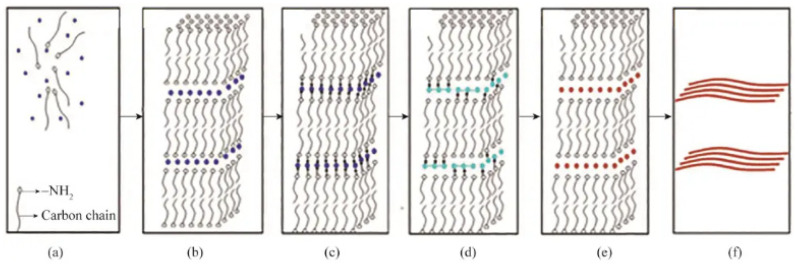
(**a**–**f**) Schematic diagram of the growth mechanism of copper nanowires prepared by solvothermal method. Reprinted from Ref. [35].

The preparation process of the solvothermal method takes place in an autoclave, making the precise determination of the preparation mechanism often tricky. In reported studies, some researchers have proposed using the self-assembled molecular structure template method to prepare metal nanowires to explain the growth phenomena of metal nanowires in the autoclave. This explanation is based on the principle of the self-assembled molecular structure template method, which uses ordered aggregates formed by surfactants (such as micelles, liquid crystals, etc.) as templates to promote the growth of metal nanowires under hydrothermal conditions. This explanation provides a possible approach to understanding the mechanism of metal nanowire preparation using the solvothermal method. For example, Zheng et al. [35] described the process of preparing copper nanowires by the solvothermal method as follows: Under low-concentration conditions, oleyl alcohol (OLA) exists in water in a random distribution (see Figure 5a). As the concentration of OLA increases, to reduce the system’s free energy, OLA molecules self-assemble into lamellar micelles by reducing the contact area between the hydrophobic tails and water. Concurrently, Cu^2+^ ions transfer from the liquid phase to the hydrophilic layer created by the OLA molecules (refer to Figure 5b). At a particular temperature, these Cu^2+^ ions combine with OLA to form complexes (as indicated in Figure 5c). OLA reduces divalent copper ions inside the micelles to monovalent copper ion complexes (see Figure 5d). Subsequently, these monovalent copper ion complexes are further reduced into copper crystals (see Figure 5e). The OLA’s “capping” action on certain faces of copper crystals induces their anisotropic development, ultimately forming copper nanowires (see Figure 5f).

### 2.4. Molecular Beam Epitaxy

**Figure 6:**
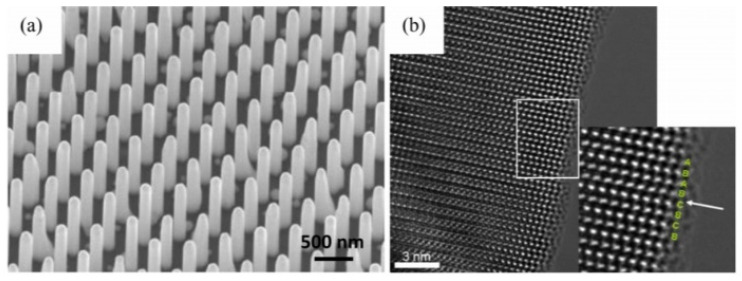
(**a**) Scanning electron microscopy (SEM) image showing a set of Au-assisted arrays of GaAs NWs [36]. Copyright 2018, AIP Publishing. (**b**) High-resolution transmission electron mi-croscopy (HRTEM) image of a single GaAs NW edge [38]. Copyright 2007, IOP Publishing.

Nanowires (NWs) are typically grown using the vapor–liquid–solid (VLS) method [36]. In this process, metal seed particles (such as Au) are in a liquid state at the growth temperature and deposit onto the surface of the solid NW from the vapor phase. The self-assisted growth method uses an element of the NW itself as the seed particle, such as Ga, to grow GaAs, which avoids introducing foreign metal elements (such as Au). Foreign metal particles located at the top of the nanowire may have adverse effects, such as contaminating the nanowire, increasing electrical contact resistance, or causing light reflection in photovoltaic devices. Although these foreign metal particles can be etched after growth or device processing, the etching process may not be simple and adversely affect device performance. The VLS method can use oxide masks to prevent growth on surfaces between NWs. By patterning techniques (such as photolithography, electron beam lithography, and nanoimprint lithography), a series of periodically arranged holes can be formed in the oxide mask, thereby exposing the substrate (e.g., Si). Metal seed particles (such as Au or Ga) form in these oxide holes, and NW growth is triggered by these metal droplets. NWs nucleate in the holes, forming a selective area (SA) epitaxy of periodically arranged NW arrays, as shown in Figure 6a. The VLS process can control the size, position, composition, impurity doping, and dimensions of semiconductor NWs [37].

**Figure 7:**
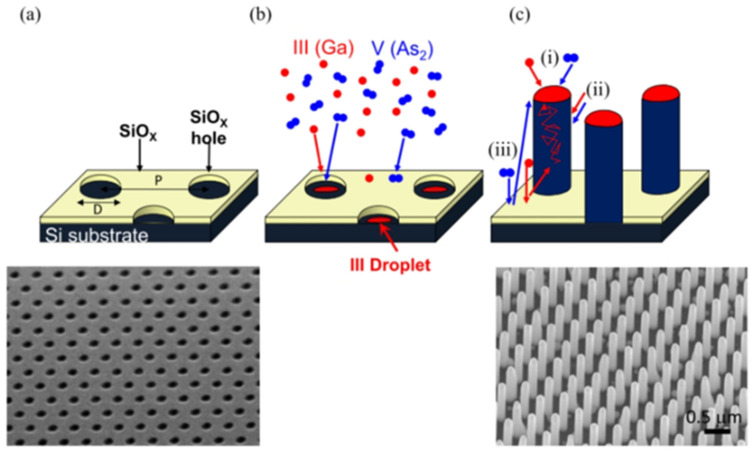
(**a**) An oxide mask (SiOx) is formed on silicon (Si), and a series of holes with a diameter of D (usually 50–100 nm) and a period of P (usually a few hundred nm) are patterned by photolithography. (**b**) Vapor phase growth of NWs. The MBE growth is described in a condition that includes group III atoms (e.g., Ga) and group V dimers (e.g., As2) in a gas phase flux. Group III droplets (e.g., Ga) are formed in the pores. (**c**) Nucleation of NWs on Ga droplets located in the wells [36]. Copyright 2018, AIP Publishing.

Molecular beam epitaxy (MBE) is a commonly used vapor deposition method for growing III-V NWs. Figure 7 illustrates the self-catalyzed and selective area MBE of NWs. As shown in Figure 7c, NW development can occur via several pathways: (1) direct collision with seed particles, (2) collision with NW side surfaces, and (3) collision with oxide masks, which is then followed by diffusion and re-release to nearby liquid droplets or side walls of other NWs. Group III elements can spread along the NW side surfaces, whereas Group V diffusion is generally minimal due to the higher vapor pressure of Group V elements. The significance of these processes is influenced by the growth technique and conditions, such as temperature, the ratio of V/III flow rates, and the rate of collision. These factors can adjust the dimensions and shape of NWs, promoting either radial or axial expansion. For example, under low-temperature MBE growth conditions (around 500 °C), radial NW growth is often preferred because the spread of Group III elements on the NW side surfaces is reduced, and nucleation on these surfaces becomes more dominant, thus encouraging radial expansion.

**Figure 8:**
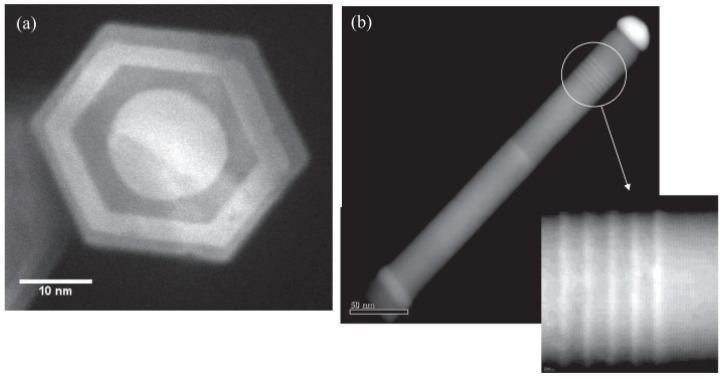
HAADF-STEM images reveal the GaAs (bright contrast) and GaP (dark contrast) regions in (**a**) a cross-sectional view of the NW core–shell structure and (**b**) an axial NW structure, with insets showing magnified views [36]. Copyright 2018, AIP Publishing.

In contrast, at high MBE growth temperatures, axial NW growth usually predominates, as atomic diffusion is sufficient for Group III atoms to reach the seed particles at the top of the NWs through diffusion along the NW sidewalls, thus promoting axial growth. Switching species during NW growth can create heterostructures, as shown in Figure 8. Finally, during the growth process, NWs can be infused with dopants to regulate their electronic characteristics, particularly for fabricating the p-n junction diodes essential in the majority of optoelectronic devices.

## 3. Nanowire Devices for Simulating Brain-like Computing

### 3.1. The Role of Nanowire Devices in Brain-like Computing

#### 3.1.1. Simulating Synaptic Characteristics

Traditional von Neumann architecture computers may not meet high-speed big data processing demands [13,39,40,41]. Therefore, there has been a widespread interest in neuromorphic computing in recent years [42,43,44,45]. Neuromorphic computing, with its low power consumption, high speed, and high precision, simulates the connection patterns between neurons and synapses in the human brain [6,46,47]. Unlike traditional computer architectures [47], it possesses parallelism and distribution characteristics, allowing for more efficient large-scale data handling. This new type of computing is also expected to reduce the frequency of redundant data processing and improve efficiency [12,48]. Artificial synaptic devices are considered an important method for simulating the functions of biological synapses [13]. NWs play a significant role in nanoelectronics and optoelectronics, contributing significantly to the advancement of NW-based synaptic devices for neuromorphic computing applications. This presentation will introduce the current research progress on NW-based synaptic memristors [49] and synaptic transistors [50] and discuss their potential applications in neuromorphic computing. At the same time, we hope these insights will promote the application of NW-based synaptic devices in neuromorphic systems [51].

Based on the device structure, NW-based synaptic devices are divided into two categories: synaptic memristors and synaptic transistors. Memristors can be fabricated in either vertical or lateral structures [48,49,50,52,53]. Memristors enable the convergence of computation and memory in a single spot, delivering benefits such as enhanced integration density and reduced energy usage [54]. The I-V hysteresis in memristors, which is crucial for data retention and manipulation, can be fine-tuned by modifying operational parameters to achieve distinct resistive states, namely the high-resistance state and the low-resistance state (LRS). Both volatile and non-volatile memristors have been extensively researched, with volatile memristors generally exhibiting a briefer retention time in the LRS.

**Figure 9:**
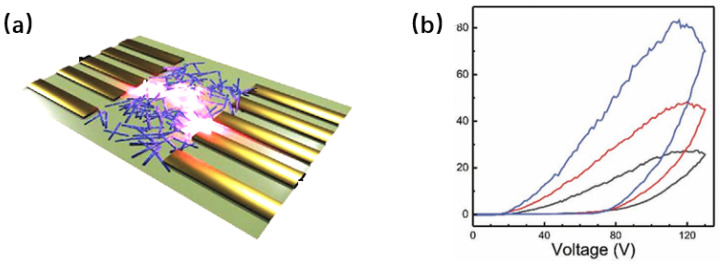
(**a**) Device structure diagram of Ag NWs coated with TiO_2_ network. (**b**) I–V curve [48]. Copyright 2020, Wiley-VCH GmbH.

In comparison, non-volatile memristors usually have a longer retention time [52]. Ni/NiO core–shell NWs are fabricated into crossbar memristors [45], where the diffusion of oxygen ions under the influence of an electric field forms or breaks conductive filaments at the center of the crossbar, leading to the observation of non-volatile resistive switching behavior. A device network based on titanium-coated Ag NWs was prepared by Li et al. [48]. The NW solution was applied to a substrate featuring pre-defined electrodes to construct the device array, as depicted in Figure 9a. The electrical properties of this network were examined, with the corresponding I-V curves displayed in Figure 9b. A pronounced current hysteresis effect is detected during the voltage sweep from 0 V to 130 V and then back.

Additionally, during continuous voltage scanning, the conductivity increases. This behavior is similar to the synaptic function of STP (short-term plasticity). When the voltage is scanned from −130 V to 130 V, resistive switching behavior can be observed in the NW device network, where the LRS (low-resistance state) is achieved through low-resistance pathways between at least two NWs. The memristive behavior of Ag has also been studied by Hosseini et al. [49] in nanowire network memristors. Synaptic functions, including learning and forgetting processes, have been mimicked by memristors. The research findings have promoted the application of NW networks in reservoir and neuromorphic computing.

**Figure 10:**
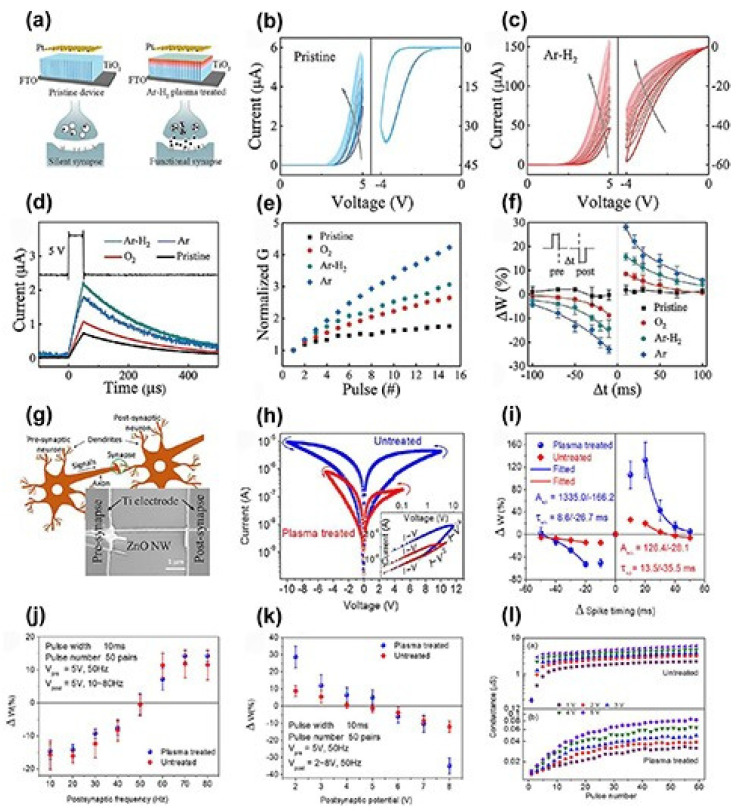
(**a**) Structure of TiO_2_ NW-based memory devices before and after plasma treatment. (**b**) I-V curve of original TiO_2_ NWs. (**c**) I-V curves of TiO_2_ NWs after Ar-H_2_ plasma treatment. (**d**) Excitatory postsynaptic current (EPSC) of devices treated with different gas plasmas. (**e**) Short-term dependent plasticity (SNDP). (**f**) STDP. (**g**) Synaptic schematic diagram and scanning electron microscopy (SEM) image of single ZnO NW memory. (**h**) I-V curve of device. (**i**) STDP. (**j**) Short-term rate-dependent plasticity (SRDP). (**k**) Short-term rate-dependent plasticity (SVDP). (**l**) Relationship between conductivity and number of pulses [47]. Copyright 2023, IOP Publishing Ltd. (**a**–**f**) [54] Copyright 2020, Wiley-VCH GmbH. (**g**–**l**) [52] Copyright 2019, IOP Publishing.

To adjust the memristive behavior of NW memristors, Shan et al. studied the synaptic plasticity of NW memristors before and after TiO_2_ plasma treatment [53]. The device structure is shown in Figure 10a, and the device was subjected to Ar-H2 plasma treatment. As shown in Figure 10b,c, for untreated TiO_2_ nanowires, the current gradually increases when scanned under positive voltage. After several scans, the current reaches saturation. When scanned under negative voltage, the change in current is negligible. This mnemonic behavior can be regarded as a silent synapse. After Ar-H_2_ plasma treatment, the current changes under both positive and negative voltages, indicating that the memory device is a functional synapse.

Furthermore, the enhancement in current following plasma treatment is significantly greater compared to that in untreated devices, likely due to an increase in oxygen vacancies post treatment. The use of plasma treatment in various gas environments has been suggested to modulate synaptic function. As illustrated in Figure 10d–f, distinct gas plasma treatments result in varying degrees of synaptic plasticity, which correlates with the oxygen vacancies created by the treatment. Similarly, Wan et al. investigated the synaptic learning mechanisms of individual ZnO nanowire memories after exposure to Ar plasma [52]. Figure 10g shows a schematic of the synapse and the SEM image of the device. According to the I-V curve of the device in Figure 10h, the operating voltage of the device after plasma treatment (5 V) is lower than that of the untreated device (10 V). This means that device power consumption can be reduced through plasma treatment. Then, the STDP, SRDP, and synaptic voltage dependence (SVDP) characteristics of the device were simulated, with the results shown in Figure 10i–k. From the STDP and SVDP results, it can be seen that plasma treatment increases the change in synaptic weight, thereby enhancing learning ability. For SRDP, the change in synaptic weight before and after plasma treatment is negligible. The dependence of conductance on the number of pulses is shown in Figure 10l. For both untreated and plasma-treated devices, the conductance increases rapidly with the number of pulses and then reaches saturation. The plasma-treated device requires more pulses to reach saturation, which also implies that learning ability is promoted by plasma treatment.

To promote the application of NW-based memristors in neuromorphic computing, it is necessary to further explore memristors with enhanced synaptic weights, high stability, and lower power consumption. In addition to plasma treatment, cation injection and laser irradiation are good options for regulating material defects. Moreover, the design of the device and material structures can also improve device performance. For example, alloying the conductive channel can significantly enhance the stability of memristors [53], and ZnO nanoparticle/CuO NW heterostructure memristors [55] can achieve excellent resistive switching behavior and synaptic plasticity. Therefore, more feasible solutions should be further explored.

**Figure 11:**
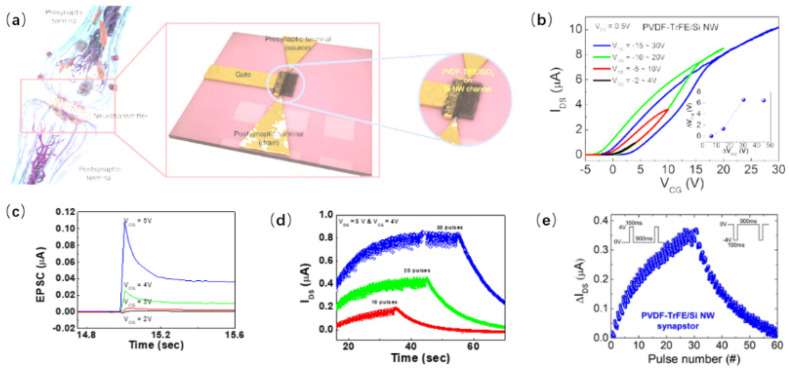
(**a**) Scheme of Si NW-based synaptic FeFET. (**b**) Transfer characteristics of Si NW FeFET with different scanning ranges. (**c**) The dependence of EPSC on pulse voltage. (**d**) SNDP. (**e**) LTP/LTD. Reprinted from Ref. [58].

Despite the advantages of low power consumption and high integration density in nanowire-based memory devices, operating memory devices on a large scale often leads to a sneak current. Additionally, memory devices face challenges in mimicking biological synapses because the memory devices’ signal transmission and learning processes will occur on the same path. There are three terminals in the device for field-effect transistors (FETs), which can achieve signal transmission and learning simultaneously. Moreover, the gate can modulate the channel conductivity [56]. Si NW ferroelectric CMOS FETs have been proven to be usable as non-volatile memory [57]. This device exhibits ultra-low power consumption. Lee et al. also demonstrated Si NW ferroelectric field-effect transistors (FeFETs) [58]. A schematic diagram of Si NW synaptic FeFET is shown in Figure 11a. Electrodes were patterned first by electron beam lithography, followed by the deposition of SiO_2_ on the device using plasma-enhanced chemical vapor deposition. Finally, a PVDF-TrFE layer was prepared by spin-coating.

**Figure 12:**
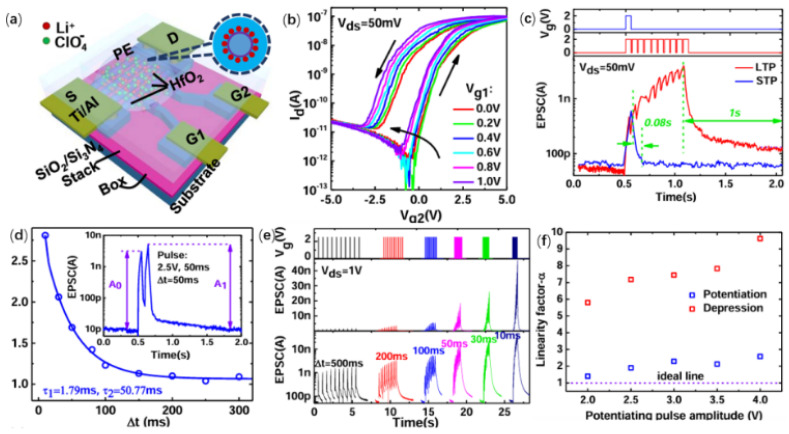
(**a**) Schematic diagram of the ionic-gated Si NW synaptic FET. (**b**) The transfer characteristics of the device under two gates modulation. (**c**) Short-term enhancement and long-term enhancement. (**d**) Paired pulse facilitation. (**e**) SRDP. (**f**) The dependence of linear factor on pulse voltage [59]. Copyright 2023, Tsinghua University Press.

Regarding the transfer characteristics of the Si NW FET, the device shows an inconspicuous hysteresis window, while the hysteresis window of the Si NW FeFET is larger. The transfer characteristics of the Si NW FeFET at different scanning ranges are shown in Figure 11b. As the scanning range increases, the hysteresis window also increases, indicating the potential of this device for application in artificial synaptic devices. The dependence of EPSC and pulse voltage was studied, as shown in Figure 12a. A transition from STP to LTP was observed as the pulse voltage increased.

Additionally, the SNDP of the device is shown in Figure 12b. As the number of pulses increases, the decay time of the current becomes longer. Figure 12c shows the potentiation and depression behavior of the Si NW FeFET under several voltage pulses. These devices display high linearity and superior symmetry, which can be enhanced by adjusting the pulse voltage. The outstanding synaptic plasticity of the Si NW FeFET renders it well-suited for applications in neuromorphic computing systems.

Ion-gated Si NW synaptic FETs have been fabricated using CMOS-compatible technology [59]. Figure 12d illustrates a schematic of the device configuration, integrating 10 parallel NWs along with two modulation gates into one unit. Under the modulation of two gate electrodes, the device’s transfer characteristics exhibit a distinct hysteresis window, with gate2 as the first variable and gate1 as the second variable, as shown in Figure 12e. These hysteresis windows can be explained by ionic migration and the dispersion of hysteresis between the nanowire and the gate electrodes. Additionally, there is a gate coupling effect between the two gates. As the voltage of gate1 increases, the threshold voltage shifts negatively, which may be related to the accumulation of additional ions. The synaptic plasticity of the device was further investigated. STP, LTP, PPF, and SRDP can all be well simulated by the device, As depicted in Figure 10h–k, the device exhibits a synaptic event power consumption of merely 375 fJ, highlighting its potential for energy-efficient artificial synapses. The device’s favorable PPF and SRDP traits position it well for decoding and transmitting information. Additionally, long-term potentiation/long-term depression (LTP/LTD) was explored, with linear factors being derived. The linear coefficient can be fine-tuned by altering the pulse voltage, signifying the gates’ effective regulation of ion accumulation.

Silicon NW-based synaptic transistors have been widely studied, including electronic FETs and optoelectronic FETs. Synaptic functions are achieved by operating NW-based synaptic devices. Researchers have proposed that neuromorphic synaptic devices must meet the following performance criteria: high linearity of conductance, a ratio of maximum to minimum conductance greater than 10, data levels greater than 32, and small variations in performance over time/between devices [60,61]. Future research should focus on exploring new NW materials and designing new NW structures to enhance NW devices’ performance further and promote NW-based synaptic devices’ application in neuromorphic computing. For instance, floating-gate transistors have outstanding advantages in data retention [62], superlattice structures are beneficial for carrier modulation and power consumption [63,64].

Recently, novel device architectures and material technologies have emerged to scale down channel lengths and enhance transistor performance without requiring extremely precise and expensive fabrication techniques. Consequently, the transistor family continues to introduce groundbreaking members, particularly vertical field-effect transistors (VFETs) [65], which stand out as one of the most promising devices offering cost-effective solutions with extreme channel length miniaturization. VFETs are fabricated by vertically stacking conductive, dielectric, and semiconductor layers, resembling the structure of stacked Schottky diodes and capacitors sharing intermediate electrodes. This design enables the channel length to be scaled down to the thickness of the active layer, achieving practical and economical miniaturization [66,67], while delivering superior current density. These characteristics make VFETs an ideal platform for a wide range of functional applications utilizing nature-inspired and bio-compound materials. For instance, Liu et al. [68] have developed multisensory artificial synapses and neural networks based on electrolyte-gated vertical organic field-effect transistors (VOFETs). By employing a crosslinking strategy, the channel length of the organic transistor was reduced to 30 nm. Owing to the ultrashort channel length and the extremely large capacitance formed at the electrolyte-channel interface, the minimum energy consumption per synaptic event was measured at 0.06 fJ, significantly lower than that of biological synapses (1–10 fJ).

Nanowire devices, while showing great potential in simulating synaptic functions, still face some key challenges and limitations. These challenges mainly include precision, efficiency, model complexity, and experimental validation. Existing simulation methods may not fully and accurately describe the physical processes in biological synapses, such as ion migration and neurotransmitter release, which could lead to discrepancies between simulation results and actual conditions. For accurate simulation, precise material and environmental parameters are needed. However, these parameters are often difficult to obtain or measure, especially at the nanoscale. Overall, future developments should focus on improving the accuracy and efficiency of models, enhancing the interpretability of models, strengthening the synergy between experiments and simulations, improving the usability and accessibility of software and tools, and promoting interdisciplinary collaboration to address the challenges of multi-scale modeling. Through these efforts, it can be expected that in the future, more accurate and practical nanowire device simulation of synaptic functions will be achieved, providing a solid foundation for the development of neuromorphic computing technology.

#### 3.1.2. Simulating Neuronal Activity

**Figure 13:**
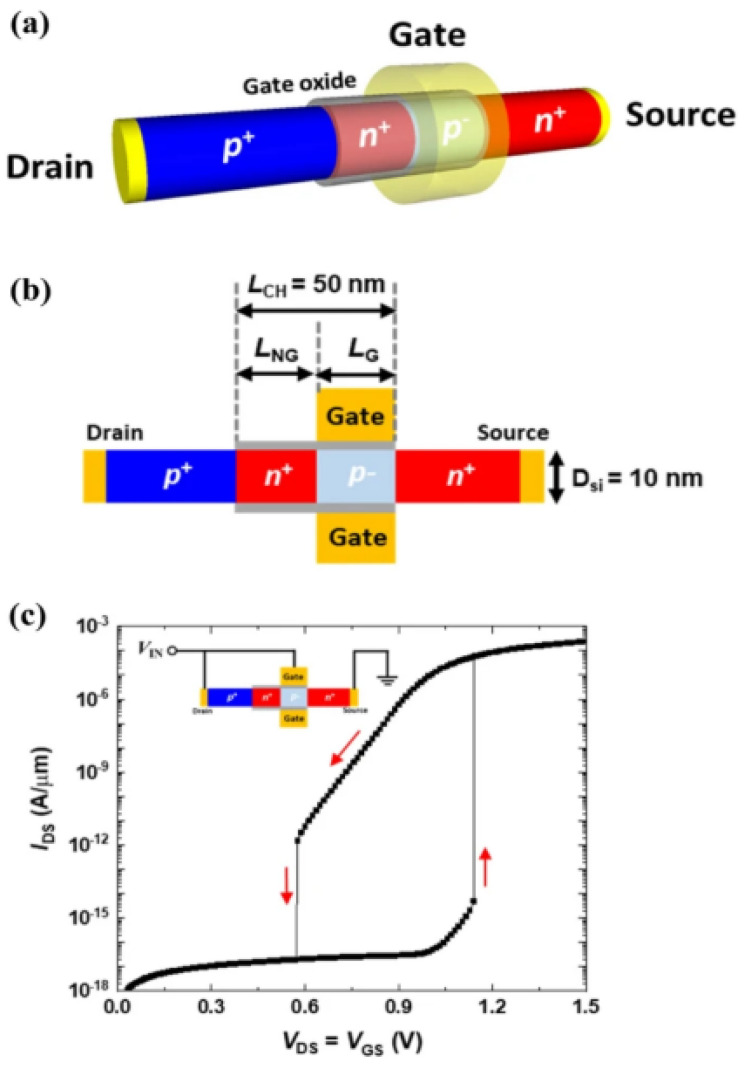
(**a**) Three-dimensional schematic of single silicon nanowire-based neuronal device. (**b**) Corresponding cross-sectional view. (**c**) Output characteristics (I–V curves) of single silicon nanowire neuronal device in gate-drain connected configuration. Reprinted from Ref. [69].

**Figure 14:**
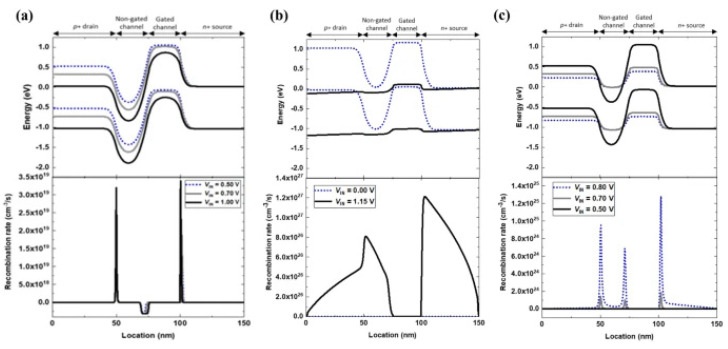
Energy band diagrams and recombination rates of single silicon nanowire neural device during integration, triggering, and reset response periods: (**a**) leakage integrated states at Vin = 0.50, 0.70, and 1.00 V, during forward scanning; (**b**) Vin state = 1.15 V; and (**c**) reset state has Vin values = 0.80, 0.70, and 0.50 V (during reverse scanning). Reprinted from Ref. [69].

The working principle and performance characteristics of silicon nanowire neuron devices are an in-depth technical field involving materials science, electronic engineering, and biological principles. The design and operation of these devices simulate the behavior of biological nervous systems, especially in signal integration and threshold triggering [69]. The fundamental functioning of a standalone silicon nanowire neuron device relies on the interplay of positive and negative feedback mechanisms within its gated p-n-p-n diode configuration. Figure 13a,b present the schematic and cross-sectional representations of such a neuron device. Figure 14 depicts the energy band diagram and recombination rate of the device, which are connected to the IDS-VDS output characteristics shown in Figure 13c. An input voltage (VIN) ranging from 0.00 to 1.00 V is applied to the gate and drain terminals to enable leakage integration within the neuron device, prior to initiating the positive feedback loop. The impact of the input voltage is bifurcated into gate and drain voltage roles, encompassing both positive and negative feedback cycles within the gated p-n-p-n diode structure.

Firstly, in the gated p-n-p-n diode structure, as the drain voltage increases, the drain and uncontrolled channel junction and the controlled channel and source junction are both forward-biased. In contrast, the uncontrolled and controlled channel junctions are more significantly reverse-biased. At the drain and uncontrolled channel junction (controlled channel and source junction), electron–hole recombination increases from 1.3 × 10^19^ (9.4 × 10^18^) to 3.2 × 10^19^ (1.5 × 10^19^), reducing the height of the junction barrier. At the uncontrolled and controlled channel junctions, electron–hole generation increases with the depletion region’s extension (≈4 nm), as shown in the bottom panel of Figure 14a. The generated charge carriers accumulate in the potential wells of the uncontrolled and controlled channels—the electrons in the conduction band of the former and the holes in the valence band of the latter (top panel of Figure 14a).

Secondly, in the gated p-n-p-n diode structure, as the gate voltage increases from 0.00 to 1.00 V, the edge of the conduction band (valence band) in the controlled channel region decreases from 1.03 to 0.37 eV (from −0.02 to −0.69 eV), allowing electrons to be injected into the potential well in the conduction band of the uncontrolled channel region [70]. The input voltage-induced barrier modulation facilitates the injection of electrons from the source into the channel area, where they gather in the potential well of the uncontrolled channel zone. This electron accumulation diminishes the barrier height, enabling the injection of holes. The accumulated holes, in turn, encourage further electron injection. As the generation of electron–hole pairs increases, the depletion region expands, affecting charge carriers’ transport and accumulation behavior. The depletion region’s expansion helps control the current flowing through the device more effectively. The charge carrier accumulation caused by the input voltage simulates the leakage integration of biological systems. The leakage integration in biological systems includes the accumulation of charge carriers: the accumulation of charge carriers caused by the input voltage simulates how biological neurons process received signals. In biological systems, neurons integrate multiple input signals, and when the total signal exceeds a certain threshold, an output signal is triggered. In terms of threshold behavior, silicon nanowire neuron devices can be designed to have a system with similar “threshold” behavior by adjusting voltage and structural parameters, which is crucial for building artificial neural networks capable of performing complex logic and decision-making.

Future research should further expand the application of NW synaptic devices in neuromorphic computing. For example, floating-gate transistors have outstanding advantages in data retention [70] and superlattice structures are beneficial for carrier modulation and power consumption [71,72].

Comparison of nanowire devices with other material-based synaptic devices. Recent studies have highlighted the advantages and limitations of NW devices compared to synaptic devices based on other nanomaterials, such as 1D carbon nanotubes (CNTs) and 2D TMDs. For instance, 1D CNT-based synaptic devices exhibit ultra-high carrier mobility and mechanical flexibility, enabling efficient signal transmission in flexible electronics. However, their integration density is limited by challenges in precise alignment and contact resistance at junctions. In contrast, NW devices achieve higher integration density through controllable synthesis (e.g., CVD or template methods) and compatibility with CMOS processes, which is critical for large-scale neuromorphic systems. 2D TMDs (e.g., MoS_2_) have shown exceptional scalability and tunable electronic properties, making them suitable for low-power synaptic transistors. Nevertheless, their fabrication often requires complex transfer processes and suffers from interfacial defects, which degrade device reliability.

The working mechanism of silicon nanowire neuron devices involves complex physical processes, especially in threshold triggering and feedback regulation of signal processing. These operations are achieved by finely controlling voltage and monitoring the behavior of charge carriers. Upon surpassing the threshold voltage of 1.14 V, the input voltage causes the accumulated charge carriers to lower the potential barrier, thereby initiating a positive feedback loop. This energy band transition is depicted at Vin = 1.15 V in Figure 14b. The positive feedback loop eliminates the potential well, leading to electron–hole recombination across the entire gated p-n-p-n diode structure, as shown in Figure 14b. Post triggering, the input voltage decreases from 1.15 V to 0.70 V, but the positive feedback persists until a negative feedback loop is activated. By adjusting the potential barrier with a diminished input voltage, electron–hole recombination is induced at various junctions, gradually rebuilding a barrier that impedes electron injection. The charge carrier recombination raises the barrier height, halting electron injection. This interplay between the potential barrier and charge carriers forms a negative feedback loop, locking the neuron device. When the input voltage falls below the lock voltage, the negative feedback loop restores the potential barrier, as illustrated in the top diagram of Figure 14c.

In contrast to the positive feedback loop that results from charge carrier accumulation in the potential well, the negative feedback loop dissipates the accumulated charge carriers due to electron–hole recombination at the junctions between the uncontrolled and controlled channels, as illustrated in Figure 14c. Silicon nanowire neuron devices achieve signal processing functions similar to biological neurons by precisely controlling voltage and monitoring internal charge dynamics. Positive and negative feedback loops enable the device to perform complex logical operations, including signal integration, threshold triggering, state maintenance, and self-reset, demonstrating its potential in advanced neuromorphic electronic devices.

The future development of nanowire devices simulating neuronal activity is an essential frontier in neuroscience and artificial intelligence. As our understanding of brain neural networks deepens, scientists constantly seek to replicate their structure and function artificially to achieve more efficient information processing systems. Overall, the future development of nanowire devices simulating neuronal activity will depend on an in-depth understanding of neuromorphic dynamics, continuous progress in simulation technology, strengthening of experimental validation, improvement of software tools, and promotion of interdisciplinary collaboration. Through these efforts, it can be expected that more accurate and practical nanowire devices simulating neuronal functions will be achieved, providing a solid foundation for developing neuromorphic computing technology.

#### 3.1.3. Nanowire Devices for Neuromorphic Computing

**Figure 15:**
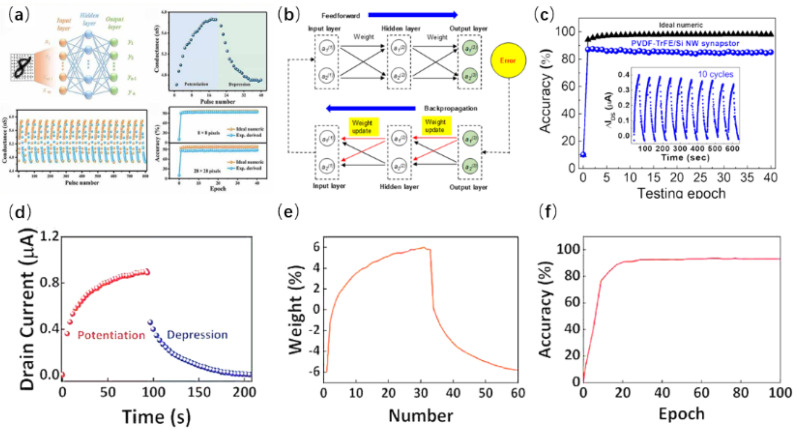
(**a**) NW synaptic memristor for pattern recognition using WO3. (**b**) The process of backpropagation algorithm. (**c**) Simulation of MNIST pattern recognition based on Si NW synaptic FeFET. (**d**) Application of LTP/LTD in ZnO NW optoelectronic synaptic transistors. (**e**) The changes in current weight during the enhancement and inhibition process of ZnO NW synaptic transistors. (**f**) Recognition accuracy [47]. Copyright 2023, IOP Publishing Ltd. (**a**) [50] Copyright 2022, AIP Publishing. (**b**,**c**) Reprinted from Ref. [58] (**d**,**f**) [86] Copyright 2021, IOP Publishing Ltd.

Neuromorphic computing is an artificial synaptic device that simulates the structure and function of the human brain. Neural networks are typically built based on artificial synaptic devices and further applied to neuromorphic computing. Pattern recognition is a typical example of neuromorphic computing [73,74]. It requires neural networks constructed with synaptic devices to recognize the typical features of digital images with high accuracy [75,76]. NW-based synaptic memristors and NW synaptic transistors have been used for pattern recognition. A WO3 NW synaptic memristor has been proposed for pattern recognition [50]. Figure 15a shows that a three-layer artificial neural network was constructed to recognize handwritten digits. The handwritten digits were divided into multiple pixels and input into the input layer. The input signals were processed in the hidden layer, and then the output layer gave the recognition results. Based on the LTP/LTD characteristics, after training for 40 cycles, the simulated recognition accuracy was 94% for 8 × 8 pixel images and dropped to 85% for 28 × 28 pixel images. Wang et al. proposed a number-analog integrated memristor based on CuO NW heterostructures [55], and the device was used to simulate the recognition of handwritten digits. Under supervised learning, the recognition accuracy reached 93%. A three-layer artificial neural network (ANN) based on the backpropagation algorithm was constructed from Si NW synaptic FeFETs [58]. Figure 15b shows that after 40 training cycles, the recognition accuracy reached 85.1% when identifying 28 × 28 pixel MNIST images.

Wang et al. [77] reported an organic electrochemical transistor (OECT) capable of integrated sensing, memory, and processing functions. As a non-volatile synaptic device, it demonstrates 1024 distinct states with wide dynamic range and state retention exceeding 10,000 s. The homogeneous integration of such devices enables the implementation of spiking neural networks (SNNs) and STDP for emulating conditioned reflex behaviors. The cv-OECT exhibits 1024 distinguishable conductance states during long-term potentiation (LTP) [78], with the bit capacity further scalable through programming pulse width/amplitude modulation. The intrinsic low conductance of on mixed ionic–electronic conductor (OMIEC) materials combined with the high transconductance of cv-OECTs ensures robust resistance to computational errors induced by read/write noise and conductance drift, even at high-conductance-state densities. Beyond LTP characteristics, the cv-OECT successfully implements biologically plausible STDP learning rules [79]. Compared to other electronic synapses [80], this design offers three key advantages: (1) precise non-volatile analog conductance modulation; (2) high off-state resistance in the T-channel that suppresses conductance drift caused by sneak gate currents [81]; (3) elimination of heterogeneous integration or complex pulse engineering requirements [82], thereby establishing foundations for building biologically credible SNNs with homogeneous architecture [83]. Most reported OECTs employ relatively large channel lengths ranging from several to tens of micrometers, which inherently limits device performance and results in low transistor density. A novel vertical OECT architecture has been developed to address these limitations, achieving nanoscale channel lengths as short as ∼100 nm. These optimized devices demonstrate exceptional performance metrics: high on-current exceeding 20 mA at a low bias voltage of 0.5 V, rapid transient response faster than 300 μs, and outstanding transconductance reaching up to 68.88 mS [84]. Denise Maria de Andrade et al. [85] reported an organic phototransistor (OPT) featuring an ultra-short conductive channel (tens of nanometers) and exceptional photoelectric conversion efficiency. The OPT is based on a vertical organic field-effect transistor (VOFET) architecture, which employs a rolled-up metallic nanomembrane (NM) as the drain electrode and a photolithographically patterned (rectangular) perforated source electrode. This design expands the conventional VOFET concept by enabling the integration of ultra-thin active layers and reliable gate-modulated control over channel current. The device exhibits low operating voltages (<5 V) and an enhanced on/off current ratio (∼10^5^). Under blue light illumination, it demonstrates remarkable photosensitivity and rapid photoelectric conversion. The core advantage of non-electrical gating characteristics lies in its ability to overcome the limitations of traditional gate-voltage control, providing neuromorphic computing systems with multimodal signal processing capabilities, such as enabling ultra-low power operation and concurrent multi-signal responsiveness.

Photoelectric synapses hold promise in emulating artificial vision and are well suited for developing artificial neural networks aimed at pattern recognition. Shen et al. introduced a ZnO NW photoelectric synaptic transistor for identifying handwritten numerals, utilizing light stimulation for the potentiation phase and electrical stimulation for the depression phase [86]. The potentiation and depression processes are shown in Figure 15d,e. Based on the device’s excellent LTP/LTD characteristics, when recognizing 16 × 16 pixel images, the recognition accuracy reached 92% after just 20 training cycles.

**Figure 16:**
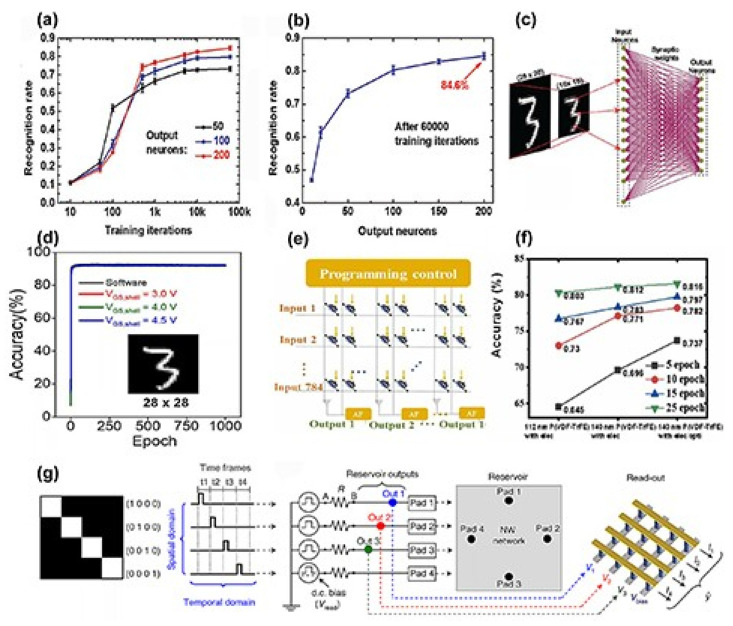
(**a**) Dependence between recognition rate and training iterations in ionic-gated Si NW synaptic FET SNN. (**b**) Dependency between recognition rate and output neurons. (**c**) Core–shell dual-gate NW synaptic transistor for HNN. (**d**) Recognition accuracy of HNN when image is 28 × 28 pixels. (**e**) Schematic diagram of single-layer perceptron HNN. (**f**) Training outcomes. (**g**) Schematic diagram of Ag NW network resistive memristive device used for reservoir calculation [47]. Copyright 2023, IOP Publishing Ltd. (**a**,**b**) [59] Copyright 2020, RSC Pub. (**c**,**d**) Reprinted from Ref. [88]. (**e**,**f**) [89] Copyright 2021, Elsevier Ltd. (**g**) Reprinted from Ref. [91].

Beyond artificial neural networks (ANNs), SNNs offer an alternative approach for pattern recognition tasks. The primary distinction between ANNs and SNNs is rooted in the nature of the input signals they process [87]. For artificial neural networks, the input signals are continuous, while in SNNs, the input signals are typically encoded as binary spike trains. Based on unsupervised learning, a pattern recognition SNN using ion-gated Si NW synaptic FETs is employed [59]. In contrast to supervised learning, unsupervised learning boasts benefits such as reduced costs and energy usage, and it is capable of learning from data without labels. However, its recognition accuracy typically falls short compared to supervised learning methods. Figure 16a,b display the pattern recognition outcomes utilizing SNNs. After undergoing 60,000 training iterations, the accuracy reached 84.6%. To enhance this accuracy, optimizing the linearity of the synapses is recommended.

Both ANNs and SNNs can be referred to as hardware neural networks (HNNs) as long as they are integrated into hardware. A simulated core–shell dual-gate NW synaptic transistor was constructed into a single-layer HNN [88]. The HNN was employed to categorize image data from the MNIST dataset, emulating the device’s capability for supervised learning. The accuracy of recognition was determined to be influenced by the gate voltage and the number of image pixels. The outcomes are presented in Figure 16c,d. When the gate voltage is 4.5 V, after 1000 training cycles, the recognition accuracy can reach as high as 92.28% for 28 × 28 pixel images and drops to 90.17% for 16 × 16 pixel images. An analog HNN for pattern recognition was constructed using P(VDF-TrFE)-coated InGaAs NW synaptic transistors [26]. After a few training sessions, the recognition accuracy can reach 80% with even lower power consumption.

Synaptic devices based on NWs can be deployed in reservoir computing applications to handle both temporal and spatial signals [89]. Self-organized Ag NW network memristive devices have been proposed for reservoir computing [90]. A schematic diagram of the reservoir computing device is shown in Figure 16g. The device exhibits nonlinear synaptic plasticity and short-term memory behavior and can recognize spatiotemporal patterns.

In addition to pattern recognition, neuromorphic computing can be used for image processing, speech recognition, and motion monitoring [91]. However, little has been studied about applying NW synaptic devices in these fields. Perovskite NW memory has been used for image processing [92]. The device’s capability for multi-level switching has enabled the successful embossing, outlining, and sharpening of images. Additionally, Ag NW nanocomposite hydrogel sensors have been developed for the detection of human movement [93]. Future research should further expand the application of NW synaptic devices in neuromorphic computing.

### 3.2. Nanowire Devices Simulate Brain-like Computing in Other Application Areas

#### 3.2.1. The Application of Nanowire Devices in Brain-like Computing in the Field of Medicine

Nanowire devices for brain-like computing have a broad range of application prospects in medicine [94], such as neural interfaces and neural repair. Nanowire arrays can be used as neural interfaces [95], transmitting electrical signals from the brain or nervous system to external devices, or vice versa, from external devices to the nervous system [96], to achieve control and repair of the nervous system. This could have great potential in dealing with neurological diseases or injuries [97], such as helping to restore motor function or alleviate chronic pain [98].

Brain–computer interfaces: Nanowires can serve as key components of brain–computer interfaces [99], playing a central role in developing brain–machine interfaces (BMIs). By providing extremely high sensitivity and selectivity, nanowires can help capture subtle neural activities and convert them into signals that external devices can understand and vice versa. This is crucial for developing high-performance prosthetics, assistive vision and hearing devices, and systems for directly controlling computers and other devices from the brain. Allowing humans to interact efficiently with computers or other external devices is significant for helping people with disabilities regain function, improve quality of life, and develop new types of assistive devices.

Neuroscientific research: In basic neuroscience research, nanowire technology can monitor and manipulate nerve cells’ activity in real-time, providing in-depth information on how the nervous system responds to stimuli. This is crucial for understanding brain function in both normal and pathological states and helps develop new treatment methods to combat neuropsychiatric diseases. Nanowire technology can provide high-resolution, high-sensitivity monitoring and measurement of nervous system function and activity, thereby promoting basic research on the brain and nervous system and helping to deeply understand the working principles of the nervous system and mechanisms related to diseases [100].

Brain disease treatment: Nanowire technology is also expected to treat brain diseases [101], such as epilepsy, Parkinson’s disease, and Alzheimer’s disease. By precisely controlling the design and function of nanowires [102] or using them for local stimulation or inhibition of specific neural circuits, targeted treatment of affected areas can be achieved, thereby improving therapeutic effects and reducing damage to healthy tissues.

Nanowire technology has shown tremendous potential in medicine and biomedical engineering [103], especially in achieving advanced neural function restoration and treatment of nervous system diseases. With this technology’s further development and application, we look forward to seeing more innovative solutions to improve human health and quality of life.

#### 3.2.2. Application of Nanowire Devices in Informatics

Nanowire devices for brain-like computing have many potential applications in the field of informatics, some of which include the following:

Neural network hardware implementation [104]: A significant breakthrough for nanowire devices in informatics is their use in constructing neural networks on hardware. By mimicking the connections and information transfer between neurons, nanowire devices can implement the functions of neural networks in a highly biomimetic manner. These artificial neural networks can directly process complex data types such as images, sound, and text at the hardware level. They can perform artificial intelligence tasks efficiently and quickly beyond traditional computer software solutions. Especially in the fields of image recognition, speech recognition, and natural language processing, nanowire neural networks have shown exceptional performance, providing strong support for real-time data processing and analysis. Nanowire devices can simulate the connections and information transfer between neurons, thus being used to construct neural networks on hardware [105,106]. This neural network hardware can implement various artificial intelligence tasks, such as image recognition, speech recognition, and natural language processing [107].

Energy efficiency optimization: With the increase in computational demands and the rise of environmental awareness, designing more energy-efficient computing systems has become an important research direction. Nanowire devices have attracted much attention due to their low power consumption and high energy efficiency. These devices can perform high-speed computations with extremely low energy consumption, making them suitable for constructing brain-inspired computing architectures. This architecture mimics the processing methods of the human brain, significantly reducing energy consumption when dealing with large-scale data. By adopting nanowire technology, future computing systems can achieve higher computation speeds and more accurate data analysis and significantly reduce operational costs, promoting the development of green computing technologies. Nanowire devices are characterized by low power consumption and high energy efficiency [108], which can be used to design more energy-efficient computing systems. By utilizing the brain-inspired computing architecture implemented with nanowire devices, significant energy savings can be achieved when processing large-scale data, thereby reducing the system’s operational costs [109].

Intelligent sensors: With the increase in computational demands and the rise of environmental awareness, designing more energy-efficient computing systems has become an important research direction [110]. Nanowire devices have attracted much attention due to their low power consumption and high energy efficiency. These devices can perform high-speed computations with extremely low energy consumption, making them suitable for constructing brain-inspired computing architectures. This architecture mimics the processing methods of the human brain, significantly reducing energy consumption when dealing with large-scale data. By adopting nanowire technology, future computing systems can achieve higher computation speeds and more accurate data analysis and greatly reduce operational costs, promoting the development of green computing technologies. Leveraging the characteristics of nanowire devices [111], intelligent sensor systems can be designed for real-time monitoring and analysis of data in the environment [112]. These intelligent sensors can be applied to the Internet of Things, smart cities, and health monitoring, among other fields, providing people with a smarter and more convenient way of life.

Adaptive computing systems: Adaptive computing systems represent the future trend of computing technology development. Brain-inspired computing systems based on nanowire devices possess adaptability, meaning they can automatically adjust their behavior and performance in response to environmental and task changes [113,114]. This adaptability makes nanowire technology highly suitable for autonomous vehicles, intelligent robots, and smart home applications. In these applications, computing systems need to be able to understand and adapt to the constantly changing external environmental conditions and users’ needs and behavioral patterns. By implementing more efficient and intelligent autonomous decision-making and actions, adaptive computing systems provide users with safer, more convenient, and personalized services [115].

These potential applications of nanowire devices in informatics demonstrate their broad functionality and profound impact. With the further development and application of this technology, we can look forward to the arrival of a new era of technology that is more intelligent and efficient [43].

## 4. Conclusions

As a new type of micro–nanoscale device, nanowire devices have shown great potential in brain-like computing. By researching and optimizing the performance and characteristics of nanowires, more efficient and faster brain-like computing processes can be achieved, and a series of exciting results have been obtained, laying a solid foundation for applying nanowire devices in brain-like computing. Firstly, nanowire devices have excellent electrical properties and tunability, which can achieve highly integrated and high-density device structures, providing the possibility for constructing complex neural networks and implementing large-scale parallel computing; secondly, the small size and low power consumption characteristics of nanowire devices also give them a huge advantage in brain-like computing, enabling faster and more energy-efficient computing processes; finally, nanowire devices have excellent nonlinear characteristics and plasticity, which can simulate the excitability and inhibitory nature of biological neurons. By controlling the structure and performance of nanowires, dynamic responses and learning capabilities similar to biological neurons can be achieved, thus realizing adaptability and intelligence in brain-like computing. This also provides new ideas and methods for constructing more intelligent brain-like computing systems.

Therefore, nanowire devices have great potential and application prospects in brain-like computing applications. Through continuous research and optimization in the future, more efficient, intelligent, and stable brain-like computing systems can be achieved, providing new solutions for artificial intelligence and neural network computing. It is believed that soon, nanowire devices will become an important part of brain-like computing, promoting the steady advancement of artificial intelligence technology development and application.
